# Genetic variant in *IL33* is associated with susceptibility to rheumatoid arthritis

**DOI:** 10.1186/ar4554

**Published:** 2014-04-29

**Authors:** Chun Li, Rong Mu, Jianping Guo, Xinyu Wu, Xin Tu, Xu Liu, Fanlei Hu, Shiwei Guo, Jiaxin Zhu, Huji Xu, Zhanguo Li

**Affiliations:** 1 Department of Rheumatology and Immunology, Peking University People’s Hospital, 11 South Xizhimen Street, Beijing 100044, China; 2 Key Laboratory of Molecular Biophysics of Ministry of Education, College of Life Science and Technology, Center for Human Genome Research, Cardio-X Institute, Huazhong University of Science and Technology, Wuhan, China; 3 Department of Rheumatology and Immunology, Shanghai Changzheng Hospital, The Second Military Medical University, Shanghai 200003, China

## Abstract

**Introduction:**

Interleukin (IL)-33 is a proinflammatory cytokine contributing to the pathogenesis of rheumatoid arthritis (RA). The gene encoding IL-33 may serve as a genetic factor and be associated with the risk of RA. To investigate the potential association between *IL33* and RA, we performed a case–control study based on Chinese Han population.

**Methods:**

A three-stage case–control study was performed. Two tag single-nucleotide polymorphisms (SNPs) (rs7044343 and rs10975514), mapping to the *IL33* gene, were first genotyped in the discovery population. We further genotyped rs7044343 and rs10975514 in the validation and replication population. The associations between the two tag SNPs and phenotypic subgroups of RA and levels of serum IL-33 were assessed with a logistic regression model.

**Results:**

In the discovery population, the CC genotype of rs7044343 was associated with RA patients (odds ratio (OR) = 0.777, 95% confidence interval (CI), 0.611 to 0.988; *P* = 0.040). After anti-citrullinated peptide antibody (ACPA) stratification, the CC genotype of rs7044343 was also shown to be a protective genotype in RA without ACPA (OR = 0.610; 95% CI, 0.379 to 0.982; *P* = 0.042). In the validation population and replication population, the association between rs7044343 and RA, especially ACPA-negative RA, was still significant. A meta-analysis of discovery, validation, and replication panels confirmed the association between CC genotype of rs7044343 and RA (*P*_combined_ = 0.0004; OR_combined_ = 0.77; 95% CI, 0.67 to 0.89). No evidence was found for heterogeneity between three sample sets (P_*het*_ = 0.99; *I*^2^ = 0%). Similar results were also obtained in ACPA-negative RA (*P*_combined_ = 0.0002; OR_combined_ = 0.57; 95% CI, 0.43 to 0.77). No association was detected between rs10975514 polymorphism and RA susceptibility in the discovery and validation population. The serum levels of IL-33 were significantly lower in the patients with the rs7044343 CC genotype.

**Conclusion:**

The CC genotype of rs7044343 in *IL33* is associated with RA patients and downregulates IL-33 expression in RA.

## Introduction

Rheumatoid arthritis (RA) is an autoimmune disease characterized by chronic inflammation of synovial joints without precisely known pathogenesis. Genetic factors contribute to the development of RA; it was estimated that the total heritability of RA is approximately 66% [1]. Current genome-wide association studies (GWASs) have identified 46 genetic loci, such as *HLA*, *PTPN22,* and *CTLA4* associated with RA [2]; however, the identified risk loci of RA have modest effect sizes (odds ratios in the range of 0.78 to 2.78) and can explain only about 16% of the RA heritability [3]. Therefore, more population-based studies are needed to find the genetic basis of RA.

Interleukin (IL)-33 was recently identified as a member of the IL-1 family and a ligand for the IL-1 family receptor ST2 [4]. In patients with RA, immunohistochemistry and *in situ* hybridization have identified IL-33 residing in the synovial cells of inflamed joints [5]. The level of IL-33 was elevated in both serum and synovial fluid and associated with autoantibody production, bone erosion, and interstitial lung disease [6,7]. In a murine model, IL-33 could exacerbate collagen-induced arthritis (CIA) and elevate the production of proinflammatory cytokines and anticollagen antibodies [8]. The ST2 antibody that blocks IL-33 signaling could attenuate the severity of CIA [9]. These studies suggest that IL-33 plays an important role in the pathogenesis of RA and indicate that the *IL33* genetic variants associated with RA merit further investigation.

## Methods

### Patients and controls

In total, 1,952 patients with established RA and 1,755 unrelated healthy controls of Northern Han Chinese origin were included in this study. The discovery population of 700 cases and 598 controls was assembled from Northern Han Chinese; the validation population of 586 cases and 456 controls was assembled from southern China; and the replication population of 666 cases and 701 controls was assembled from both northern and southern China. All patients fulfilled the revised criteria of the American College of Rheumatology for RA [10]. The healthy control subjects were defined as healthy individuals without inflammatory arthritis by medical history, general examinations, and laboratory examinations and were individually matched to RA cases on the basis of sex, ethnicity, and local residential region. The study was approved by the ethics committee of Peking University People’s Hospital (FWA00001384), and informed consents were obtained from all participants.

After oral and written informed consents had been obtained, genomic DNA and serum samples were extracted from peripheral blood of patients and healthy controls. The following clinical data were recorded for ascertainment of the clinical phenotype of RA patients: age at RA onset, sex, disease duration, and rheumatoid factor (RF) and ACPA status.

### Genotyping methods

**Figure 1:**
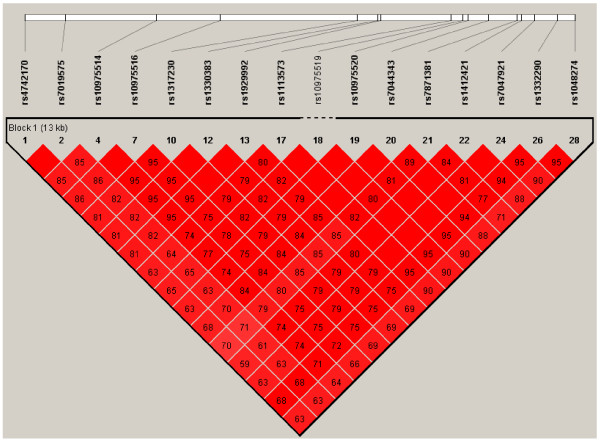
**Information of *IL33 *SNPs from HapMap CHB database.** All *IL33* SNPs are in one block and in high linkage disequilibrium (LD) (D’ > 0.92).The numbers present the *r*^2^ between the SNPs. Two SNPs (rs7044343 and rs10975514) were selected in this study. SNP, single-nucleotide polymorphism; CHB, Han Chinese in Beijing.

Haplotypes from the HapMap database (Han Chinese in Beijing, CHB) [11] were used to select tag SNPs (*r*^2^ = 0.8) residing in *IL33* (dbSNP data (build129)). In total, 16 SNPs were identified in CHB. All SNPs were in one block and in high linkage disequilibrium (LD) (D’ > 0.92; Figure 1). Tag SNPs were selected according to the following principles: (a) linkage disequilibrium (LD) between SNPs according to Haploview (v. 4.2) based on HapMap CHB data sets with the thresholds of *r*^2^ > 0.8 and D’ > 0.7 to reduce the redundancy; (b) potential functional sites predicted by bioinformatics (Promoter and Genevar); and (c) a minor allele frequency (MAF) threshold of >0.05. Data were excluded if the allele call rate was <95%.

Genotyping of rs7044343 and 10975514 was performed by using predesigned Taqman SNP Genotyping Assays (C_31083545_10; Applied Biosystems, Foster City, CA, USA). Allelic discrimination was performed in an ABI 7300 Real-Time PCR system (Applied Biosystems). The successful genotyping rate was 99.0%.

The genotypes obtained were subsequently validated and confirmed by sequencing the PCR products with an ABI 3700 automated sequencer (Applied Biosystems). In brief, 130 individuals of cases and controls were sequenced for confirmation of the rs7044343 and rs10975514. The results of DNA sequence analysis are shown in Additional file 1: Figure S1. The confirmation rate was 100%.

Meta-analysis was performed by using Review Manager 5 software and carried out with the Mantel-Haenszel method. A significant *I*^2^ statistic (*I*^2^ > 30%; *P* < 0.05) indicated heterogeneity for OR across studies. The fixed-effects model was applied in current meta-analyses.

Two genetic markers selected for the study, rs10975514 and rs7044343, were genotyped in the discovery population. Then we further genotyped rs7044343 and 10975514 in the validation population and replication population.

### Assay for serum levels of IL-33

Serum IL-33 was measured with enzyme-linked immunosorbent assay (ELISA), according to the manufacturer’s instructions (R&D Systems, Minneapolis, MN, USA).

### Statistical analyses

Allele frequencies were calculated and tested for agreement with Hardy-Weinberg equilibrium (HWE) by using a χ^2^ goodness-of-fit test. Differences in allele frequencies and genotype distribution of the polymorphism between RA cases and control subjects were compared by using a 2 × 2 contingency χ^2^ test with 1 degree of freedom, and calculated odds ratios (ORs) with 95% confidence intervals (CIs), adjusting for age and sex. Associations between genetic variations and susceptibility or phenotype features of RA were analyzed by using the Mann–Whitney *U* test. All statistical analyses were conducted by using SPSS 16.0 (SPSS Inc., Chicago, IL, USA). A *P* value of <0.05 was considered statistically significant in the expression of IL-33.

## Results

### Population characteristics

The demographic distribution of the population under study is shown in Table 1. The mean age in the case group was 53.6 ± 13.3 years, and 81.3% were women. The mean age at disease onset was 46.4 ± 15.6 years. The positive rates of rheumatoid factor (RF) and ACPA were 81.2% and 72.5%, respectively. The mean age of healthy controls was 40.4 ± 10.3 years, and 80.6% were women (Table 1).

**Table 1:** Clinical characteristics of the study cohorts

	**Discovery population**	**Validation population**	**Replication population**
Number of cases	700	586	666
Ethnicity	Northern Han Chinese	Southern Han Chinese	Northern and Southern
Female, %	79.9	81.2	82.8
Age, mean ± SD years	54.0 ± 13.1	52.4 ± 13.9	54.3 ± 12.8
Age at RA onset, mean ± SD years	46.4 ± 16.0	46.3 ± 15.0	46.6 ± 15.4
Disease duration, mean ± SD years	8.1 ± 8.1	7.8 ± 7.3	9.0 ± 10.3
ACPA status, % positive	72.6	75.3	68.9
RF status, % positive	81.6	78.3	84.0

ACPA*,* anti-citrullinated proteins antibodies; RF, rheumatoid factor.

### Association analysis of rs7044343 with RA

In HapMap CHB (data resulted from sequencing of 42 individuals), the allele distributions for rs7044343 T and C were 0.526 and 0.474 [11]. In our study, we found the allele frequencies for rs7044343 T and C were 0.452 and 0.548 in the control subjects (*n* = 1,755). Based on the assumption of a population prevalence of disease of 0.2%, α = 0.05, minor allele frequencies of 0.452, and linkage disequilibrium of *r*^2^ = 0.8, the study had 99.7% power to detect an additive association with an additive OR of 1.26.

Allelic and genotype frequencies of rs7044343 SNP in RA patients and controls are shown in Tables 2, 3, and 4. In the discovery population, the CC genotype of rs7044343 was associated with (OR = 0.777; 95% CI, 0.611 to 0.988; *P* = 0.040) RA patients. When we divided the RA patients into different subgroups by ACPA and RF status, the results showed that the CC genotype of rs7044343 was associated with RA patients without ACPA (OR = 0.610; 95% CI, 0.379 to 0.982; *P* = 0.042). No association was found between rs7044343 and RA after gender stratification.

**Table 2:** Association analysis of rs7044343 with RA in the discovery population

**Characteristic ( *n *)**	**Allele frequency (%)**		**Genotype frequency (%)**			**T versus C**	**CC versus TT + TC**	**CC + TC versus TT**
	**T**	**C**	**TT**	**TC**	**CC**	**OR (95% CI)**	**OR (95% CI)**	**OR (95% CI)**
						***P *** **value**	***P *** **value**	***P *** **value**
Control (598)	545 (45.6)	651 (54.4)	137 (22.9)	271 (45.3)	190 (31.8)			
RA (700)	641 (45.8)	759 (54.2)	127 (18.1)	387 (55.3)	186 (26.6)	1.009	0.777	1.354
						(0.864-1.178)	(0.611-0.988)	(1.033-1.774)
						0.912	0.040*	0.028*
RF + (418)	377 (45.1)	459 (54.9)	78 (18.7)	221 (52.9)	119 (28.5)	0.981	0.855	1.308
						(0.822-1.172)	(0.650-1.123)	(0.958-1.785)
						0.833	0.260	0.091
RF- (94)	86 (45.7)	102 (54.3)	17 (18.1)	52 (55.3)	25 (26.6)	1.007	0.778	1.359
						(0.740-1.371)	(0.477-1.269)	(0.777-2.376)
						0.964	0.314	0.282
ACP (300)	281 (46.8)	319 (53.2)	62 (20.7)	157 (52.3)	81 (27.0)	1.052	0.794	1.152
						(0.864-1.281)	(0.584-1.080)	(0.821-1.615)
						0.612	0.142	0.413
ACPA - (113)	108 (47.8)	118 (52.2)	20 (17.7)	68 (60.2)	25 (22.1)	1.093	0.610	1.395
						(0.822-1.453)	(0.379-0.982)	(0.830-2.345)
						0.539	0.042*	0.209
Female control (465)	432 (46.5)	498 (53.5)	106 (22.8)	220 (47.3)	139 (29.9)			
Female RA (482)	426 (44.2)	538 (55.8)	85 (17.6)	256 (53.1)	141 (29.3)	0.913	0.970	1.396
						(0.762-1.094)	(0.734-1.282)	(1.015-1.920)
						0.323	0.829	0.040
Female RF+ (333)	300 (45.0)	366 (55.0)	62 (18.6)	176 (52.9)	95 (28.5)	0.945	0.936	1.306
						(0.774-1.154)	(0.687-1.276)	(0.920-1.855)
						0.578	0.676	0.135
Female RF- (75)	64 (42.7)	86 (57.3)	12 (16.0)	40 (53.3)	23 (30.7)	0.858	1.037	1.569
						(0.606-1.215)	(0.611-1.761)	(0.816-3.018)
						0.388	0.892	0.177
Female ACPA+ (243)	225 (46.3)	261 (53.7)	50 (20.6)	125 (51.4)	68 (28.0)	0.994	0.911	1.154
						(0.798-1.238)	(0.647-1.285)	(0.790-1.685)
						0.956	0.596	0.459
Female ACPA- (84)	77 (45.8)	91 (54.2)	15 (17.9)	47 (56.0)	22 (26.2)	0.975	0.832	1.375
						(0.701-1.356)	(0.492-1.407)	(0.756-2.502)
						0.882	0.493	0.297
Male CON (105)	99 (47.1)	111 (52.9)	26 (24.8)	47 (44.8)	32 (30.5)			
Male RA (121)	116 (47.9)	126 (52.1)	24 (19.8)	68 (56.2)	29 (24.0)	1.032	0.719	1.330
						(0.713-1.495)	(0.399-1.296)	(0.709-2.496)
						0.867	0.272	0.374
Male RF+ (84)	75 (44.6)	93 (55.4)	15 (17.9)	45 (53.6)	24 (28.6)	0.904	0.913	1.514
						(0.602-1.359)	(0.486-1.713)	(0.742-3.088)
						0.628	0.776	0.254
Male RF- (19)	22 (57.9)	16 (42.1)	5 (26.3)	12 (63.2)	2 (10.5)	1.542	0.268	0.922
						(0.767-3.100)	(0.059-1.231)	(0.303-2.805)
						0.222	0.090	0.886
Male ACPA+ (57)	56 (49.1)	58 (50.9)	12 (21.1)	32 (56.1)	13 (22.8)	1.083	0.674	1.234
						(0.686-1.709)	(0.320-1.420)	(0.568-2.681)
						0.733	0.300	0.595
Male ACPA- (27)	29 (53.7)	25 (46.3)	5 (18.5)	19 (70.4)	3 (11.1)	1.301	0.285	1.448
						(0.714-2.369)	(0.080-1.016)	(0.498-4.211)
						0.390	0.053	0.497

*P < 0.05 versus controls; ACPAs*,* anti-citrullinated proteins antibodies; CI*,* confidence interval.

CON*,* healthy control; OR*,* odds ratio; RA*,* rheumatoid arthritis; RF, rheumatoid factor.

**Table 3:** Association analysis of rs7044343 with RA in the validation population

**Characteristic ( *n *)**	**Allele frequency (%)**		**Genotype frequency (%)**			**T vs. C**	**CC vs. TT + TC**	**CC + TC vs. TT**
	**T**	**C**	**TT**	**TC**	**CC**	**OR (95% CI)**	**OR (95% CI)**	**OR (95% CI)**
						***P *** **value**	***P *** **value**	***P *** **value**
Control (456)	409 (44.8)	503 (55.2)	100 (21.9)	209 (45.8)	147 (32.2)			
RA (586)	548 (46.8)	624 (53.2)	118 (20.1)	312 (53.2)	156 (26.6)	1.080	0.763	1.114
						(0.908-1.285)	(0.583-0.998)	(0.825-1.504)
						0.385	0.048*	0.480
RF + (354)	322 (45.5)	386 (54.5)	67 (18.9)	188 (53.1)	99 (28.0)	1.026	0.816	1.203
						(0.842-1.250)	(0.602-1.106)	(0.851-1.701)
						0.799	0.190	0.295
RF- (98)	100 (51.0)	96 (49.0)	25 (25.5)	50 (51.0)	23 (23.5)	1.281	0.645	0.820
						(0.941-1.745)	(0.388-1.070)	(0.495-1.360)
						0.116	0.089	0.442
ACPA + (281)	257 (45.7)	305 (54.3)	51 (18.1)	155 (55.2)	75 (26.7)	1.036	0.765	1.267
						(0.839-1.280)	(0.551-1.064)	(0.870-1.845)
						0.741	0.111	0.217
ACPA- (92)	94 (51.1)	90 (48.9)	21 (22.8)	52 (56.5)	19 (20.7)	1.284	0.547	0.950
						(0.935-1.764)	(0.318-0.940)	(0.556-1.622)
						0.121	0.029*	0.850
Female control (363)	319 (43.9)	407 (56.1)	75 (20.7)	169 (46.6)	119 (32.8)			
Female RA (441)	413 (46.8)	469 (53.2)	87 (19.7)	239 (54.2)	115 (26.1)	1.124	0.723	1.060
						(0.922-1.369)	(0.533-0.982)	(0.750-1.497)
						0.247	0.038	0.743
Female RF+ (291)	264 (45.4)	318 (54.6)	53 (18.2)	158 (54.3)	80 (27.5)	1.059	0.777	1.169
						(0.851-1.319)	(0.554-1.090)	(0.791-2.730)
						0.607	0.144	0.433
Female RF- (75)	77 (51.3)	73 (48.7)	20 (26.7)	37 (49.3)	18 (24.0)	1.346	0.648	0.716
						(0.946-1.914)	(0.365-1.149)	(0.404-1.268)
						0.098	0.137	0.252
Female ACPA+ (234)	217 (46.4)	251 (53.6)	43 (18.4)	131 (56.0)	60 (25.6)	1.103	0.707	1.157
						(0.873-1.393)	(0.490-1.020)	(0.762-1.755)
						0.410	0.064	0.494
Female ACPA- (73)	74 (50.7)	72 (49.3)	18 (24.7)	38 (52.1)	17 (23.3)	1.311	0.622	0.796
						(0.919-1.872)	(0.347-1.118)	(0.441-1.435)
						0.135	0.112	0.448
Male CON (89)	86 (48.3)	92 (51.7)	24 (27.0)	38 (42.7)	27 (30.3)			
Male RA (102)	98 (48.0)	106 (52.0)	23 (22.5)	52 (51.0)	27 (26.5)	0.956	0.827	1.268
						(0.615-1.486)	(0.440-1.553)	(0.656-2. 452)
						0.840	0.554	0.480
Male RF+ (63)	58 (46.0)	68 (54.0)	14 (22.2)	30 (47.6)	19 (30.2)	0.827	0.992	1.292
						(0.527-1.299)	(0.491-2.002)	(0.607-2.753)
						0.410	0.981	0.506
Male RF- (22)	23 (52.3)	21 (47.7)	5 (22.7)	13 (59.1)	4 (18.2)	1.172	0.510	1.255
						(0.605-2.268)	(0.158-1.651)	(0.417-3.777)
						0.638	0.261	0.686
Male ACPA+ (47)	40 (42.6)	54 (57.4)	8 (17.0)	24 (51.1)	15 (31.9)	0.792	1.076	1.800
						(0.479-1.311)	(0.502-2.306)	(0.737-4.397)
						0.365	0.850	0.197
Male ACPA- (18)	19 (52.8)	17 (47.2)	3 (16.7)	13 (72.2)	2 (11.1)	0.376	0.287	1.846
						(0.207-0.686)	(0.062-1.336)	(0.491-6.946)
						0.001	0.112	0.364

*P < 0.05 versus controls; ACPA anti-citrullinated proteins antibodies; CI confidence interval CON healthy controls; OR Odds ratio RA rheumatoid arthritis; RF rheumatoid factor.

**Table 4:** Association analysis of rs7044343 with RA in the replication population

**Characteristic ( *n *)**	**Allele frequency (%)**		**Genotype frequency (%)**			**T vs. C**	**CC vs. TT + TC**	**CC + TC vs. TT**
	**T**	**C**	**TT**	**TC**	**CC**	**OR (95% CI)**	**OR (95% CI)**	**OR (95% CI)**
						***P*-value**	***P*.-value**	***P*-value**
Control (701)	631 (45.0)	771 (55.0)	154 (22.0)	323 (46.1)	224 (32.0)			
RA (666)	632 (47.4)	700 (52.6)	144 (21.6)	344 (51.7)	178 (26.7)	1.103	0.777	1.021
						(0.949-1.282)	(0.615-0.981)	(0.789-1.320)
						0.201	0.034*	0.877
RF + (325)	300 (46.2)	350 (53.8)	73 (22.5)	154 (47.4)	98 (30.2)	1.047	0.919	0.972
						(0.869-1.262)	(0.691-1.223)	(0.709-1.333)
						0.627	0.563	0.860
RF-(62)	54 (43.5)	70 (56.5)	10 (16.1)	34 (54.8)	18 (29.0)	0.943	0.871	1.464
						(0.651-1.365)	(0.492-1.542)	(0.727-2.948)
						0.754	0.636	0.286
ACPA + (224)	192 (42.9)	256 (57.1)	39 (17.4)	114 (50.9)	71 (31.7)	0.916	0.988	1.335
						(0.739-1.136)	(0.715-1.365)	(0.905-1.970)
						0.425	0.943	0.2145
ACPA-(101)	103 (51.0)	99 (49.0)	23 (22.8)	57 (56.4)	21 (20.8)	1.271	0.559	0.955
						(0.946-1.708)	(0.337-0.927)	(0.580-1.571)
						0.111	0.024*	0.856
Female Control (519)	474 (45.7)	564 (54.3)	114 (22.0)	246 (47.4)	159 (30.6)			
Female RA (458)	410 (44.8)	506 (55.2)	92 (20.1)	226 (49.3)	140 (30.6)	1.040	0.997	1.120
						(0.869-1.246)	(0.759-1.309)	(0.822-1.525)
						0.668	0.982	0.473
Female RF+ (256)	237 (46.3)	275 (53.7)	56 (21.9)	125 (48.8)	75 (29.3)	1.025	0.938	1.005
						(0.829-1.268)	(0.676-1.302)	(0.700-1.444)
						0.817	0.703	0.977
Female RF- (53)	46 (43.4)	60 (56.6)	8 (15.1)	30 (56.6)	15 (28.3)	0.912	0.894	1.583
						(0.610-1.365)	(0.478-1.672)	(0.726-3.455)
						0.655	0.725	0.248
Female ACPA+ (158)	133 (42.1)	183 (57.9)	27 (17.1)	79 (50.0)	52 (32.9)	0.865	1.111	1.366
						(0.670-1.115)	(0.759-1.625)	(0.859-2.171)
						0.263	0.589	0.187
Female ACPA- (76)	72 (47.4)	80 (52.6)	14 (18.4)	44 (57.9)	18 (23.7)	1.071	0.703	1.247
						(0.761-1.506)	(0.401-1.231)	(0.673-2.308)
						0.694	0.217	0.483
Male CON (130)	125 (48.1)	135 (51.9)	30 (23.1)	65 (50.0)	35 (26.9)			
Male RA (95)	75 (39.5)	115 (60.5)	16 (16.8)	43 (45.3)	36 (37.9)	0.704	1.656	1.481
						(0.482-1.029)	(0.939-2.921)	(0.754-2. 908)
						0.070	0.081	0.254
Male RF+ (54)	42 (38.9)	66 (61.1)	10 (18.5)	22 (40.7)	22 (40.7)	0.687	1.866	1.320
						(0.435-1.085)	(0.958-3.636)	(0.594-2.934)
						0.107	0.067	0.496
Male RF- (9)	8 (44.4)	10 (55.6)	2 (22.2)	4 (44.4)	3 (33.3)	0.864	1.357	1.050
						(0.330-2.259)	(0.322-5.723)	(0.207-5.325)
						0.765	0.677	0.953
Male ACPA+ (44)	29 (33.0)	59 (67.0)	4 (9.1)	21 (47.7)	19 (43.2)	0.531	2.063	3.000
						(0.320-0.881)	(1.013-4.202)	(0. 993–9.065)
						0.014	0.046	0.052
Male ACPA- (11)	13 (59.1)	9 (40.9)	5 (45.5)	3 (27.3)	3 (27.3)	1.560	1.018	0.360
						(0.644-3.776)	(0.255-4.055)	(0.103-1.263)
						0.321	0.980	0.111

*P < 0.05 *vs* controls; *RA* rheumatoid arthritis; *CON* healthy controls; *RF* rheumatoid factor; *ACPA* anti-citrullinated proteins antibodies; *OR* Odds ratio; *CI* confidence interval.

**Figure 2:**
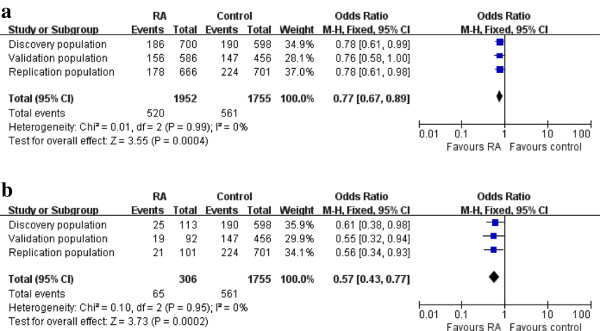
**The allele model meta-analysis for rs7044343.** A meta-analysis of discovery, validation, and replication panels confirmed the association between the CC genotype of rs7044343 and RA (*P*_combined_ = 0.0004; OR_combined_ = 0.77; 95% CI, 0.67 to 0.89). No evidence was found for heterogeneity between three sample sets (P_*het*_ = 0.99; *I*^2^ = 0) **(a)**. Similar results were also obtained in ACPA-negative RA (*P*_combined_ = 0.0002; OR_combined_ = 0.57; 95% CI, 0.43 to 0.77) **(b)**.

In the validation population and replication population, the associations between rs7044343 and RA, especially ACPA-negative RA, were still significant (OR = 0.763; 95% CI, 0.583 to 0.998; *P* = 0.048; OR = 0.547, 95% CI 0.318-0.940, *P* = 0.029; OR = 0.777, 95% CI 0.615 to 0.981, *P* = 0.034; OR = 0.559, 95% CI, 0.337 to 0.927, *P* = 0.024). A meta-analysis of discovery, validation, and replication panels confirmed the association between CC genotype of rs7044343 and RA (*P*_combined_ = 0.0004; OR_combined_ = 0.77; 95% CI, 0.67 to -0.89). No evidence was noted for heterogeneity between the three sample sets (P_*het*_ = 0.99; *I*^2^ = 0). Similar results were also obtained in ACPA-negative RA (*P*_combined_ = 0.0002; OR_combined_ = 0.57; 95% CI, 0.43 to 0.77) (Figure 2). No association was detected between rs10975514 polymorphism and RA susceptibility in the discovery and validation population (see Additional file 2: Table S1, Table S2, and Table S3).

No association was found between rs7044343 and erythrocyte sedimentation rate (ESR), C-reactive protein (CRP), or 28-joint-count disease activity score (DAS28) in RA (data not shown).

### Patients with CC genotype of rs7044343 display lower IL-33 serum levels

**Figure 3:**
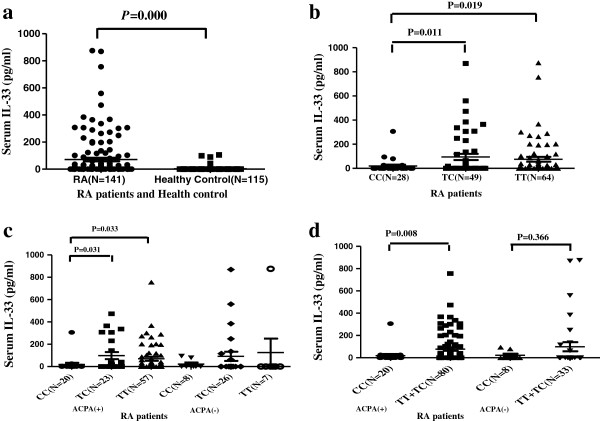
**Comparison of serum IL-33 levels among *IL33 *genotypes.** The IL-33 levels were tested in 141 DMARDs-naïve RA patients and 115 healthy controls (HCs). Serum IL-33 concentrations were significantly higher in patients with RA than in healthy controls (*P* = 0.000 **(a)**. Patients with the CC genotype showed significantly lower serum IL-33 levels than did patients with TC and TT genotypes **(b)**. When we divided the patients into ACPA-positive and ACPA-negative groups, the same result was obtained in the ACPA-positive group **(c)**. In the ACPA-negative group (*n* = 41), patients with the CC genotype also showed lower serum IL-33 levels than did patients with TC and TT genotypes (21.8 ± 40.5 versus 91.5 ± 209.5 versus 125.1 ± 331.0 ng/ml); however, no statistical difference was found. IL-33 expression was significantly higher in the dominant model (TT + TC) (*P* = 0.008 **(d)**. TT, TC, and CC represent genotypes of *IL33* rs7044343.

To evaluate whether *IL33* polymorphism is associated with dysregulation of IL-33 at the protein level, we conducted ELISA assays of 141 sera of DMARDs-naïve RA patients. The results showed that serum levels of IL-33 were significantly lower in RA patients with the rs7044343 CC genotype (*n* = 28) compared with the patients with rs7044343 TT and TC genotypes (Figure 3b), indicating that the CC genotype of rs7044343 downregulates IL-33 expression in RA. When we divided the patients into ACPA-positive and -negative groups, the same result was obtained in the ACPA-positive group (Figure 3c). In the ACPA-negative group, patients with the CC genotype also showed lower serum IL-33 level than did patients with TC and TT genotypes (21.8 ± 40.5 versus 91.5 ± 209.5 versus 125.1 ± 331.0 ng/ml); however, no statistical difference was found. Moreover, IL-33 expression was significantly higher in the dominant model (TT + TC) (*P* = 0.008; Figure 3d).

## Discussion

In the present study, we demonstrated that the CC genotype of rs7044343 in *IL33* is associated with RA patients, especially in the ACPA-negative subset. Results also showed that the CC genotype of rs7044343 was associated with lower serum IL-33 levels. Genetic factors contribute to the development of ACPA-negative RA as much as ACPA-positive RA [12]. Only a few susceptibility alleles of HLA and non-HLA genes have been associated with ACPA-negative RA [13]. From the present data, gene polymorphism of *IL33* is a new marker related to susceptibility to ACPA-negative RA.

The relation between the genotype of *IL33* and the production of autoantibodies is worth studying. Based on the present study, the protective role provided by the CC genotype of rs7044343 in RA patients may be due to the impaired expression of IL-33 protein. The mechanisms by which IL-33 is associated with RA and its specific antibodies production are still obscure. It was deduced that IL-33 drove production of Th2-associated cytokines, including IL-5 and IL-13, which could promote B-cell function, such as autoantibody production [4]. IL-33 may also contribute to the antibody production by inducing mast cell activation [6].

Genetic variants in *IL33* were reported to be associated with decreased risk of Alzheimer disease (AD) [14,15]. Chapuis *et al.*[14] showed that three intronic rs1157505, rs11792633, and rs7044343 SNPs within *IL33* decrease the risk of AD. In this study, we found that a tag SNP-rs7044343 in *IL33* was also associated with RA. This indicated that rs7044343 in *IL33* decreased the risks of both RA and AD. The inverse relation between AD and RA was challenged. Simmons *et al.*[16] found that 17 alleles associated with increased RA risk were not associated with reduced AD risk, and three RA-associated SNPs increased the risk of AD, indicating that RA genetics did not underlie the inverse relation between RA and AD, but rather may promote AD [17].

GWAS studies have shown that rs1342326 [16], rs3939286 [18], and rs2381416 [19] SNPs within *IL33* seem to be an asthma-susceptibility gene. *IL33* encodes a cytokine belonging to the IL1 superfamily, and is the natural ligand for the IL1RL1 receptor [4], which has been previously implicated in asthma, inflammation, and a number of immune disorders [20].

The present study suggests a lack of association between *IL33* polymorphism and RA disease activity index (including DAS 28 score, acute-phase reactant, and so on), although the *IL33* 7044343 CC genotype was associated with lower serum IL-33 levels. Whether the serum levels of IL-33 were related to RA disease activity shows conflicting evidence. Matsuyama *et al*. [21] reported that serum IL-33 levels were significantly higher in RA patients and correlated with disease activity. However, we found that serum IL-33 levels were not correlated to inflammation markers, such as ESR and CRP, but to autoantibodies in RA patients, which was consistent with the present finding.

Some limitations exist in our study. First, a larger sample size in different populations, especially in other ethnic populations, is required to validate the results. Second, although the results showed that the rs7044343 CC genotype of *IL33* is associated with lower serum IL-33 levels, more large-scale population study and functional experiments are needed to elucidate the association. Third, the rs7044343-polymorphism genotype distribution was not in accordance with the HWE in RA patients and discovery controls. Theoretically, cases do not need to be in HWE [22]. Rs7044343 was not in HWE in discovery controls, but was in HWE in validation and replication. This might be due to the smaller sample size or to other uncertain factors. We sequenced 130 cases and controls, and double checked the primer and probe sequences of SNPs and genotype plots of TaqMan to confirm that no genotyping bias existed.

## Conclusion

To our knowledge, we first found that the rs7044343 in *IL33* is associated with RA patients, and the CC genotype of rs7044343 is associated with lower serum IL-33 levels. These findings, together with our previous studies, suggest that IL-33, both in gene and in protein levels, plays an important role in the pathogenesis of RA.

## Abbreviations

ACPA: Anti-citrullinated peptide antibody; AD: Alzheimer disease; CHB: Han Chinese in Beijing; CI: confidence interval; CIA: collagen-induced arthritis; CRP: C-reactive protein; CTLA: cytotoxic T-lymphocyte antigen; DAS28: 28-joint-count disease activity score; DMARD: disease-modifying antirheumatic drug; DNA: deoxyribonucleic acid; ELISA: enzyme-linked immunosorbent assay; ESR: erythrocyte sedimentation rate; GWAS: genome-wide association study; HLA: human leukocyte antigen; HWE: Hardy–Weinberg equilibrium; IL: interleukin; LD: linkage disequilibrium; MAF: minor allele frequency; OR: odds ratio; PCR: polymerase chain reaction; PTPN22: protein tyrosine phosphatase N22; RA: rheumatoid arthritis; RF: rheumatoid factor; SNP: single-nucleotide polymorphism.

## Competing interests

The authors declare that they have no competing interests.

## Authors’ contributions

CL, data collection and analysis, manuscript writing, and final approval of the manuscript. RM, conception and design, critical revision, final approval, and responsibility for the manuscript. JPG, analysis and interpretation, critical revision, and final approval of the manuscript. XYW, conception and design and data collection, analysis, and interpretation, manuscript writing, and final approval of the manuscript. XT, analysis, interpretation, critical revision, and final approval of the manuscript. XL, data collection and interpretation, manuscript writing, and final approval of the manuscript. FLH, data collection and critical revision, manuscript writing, and final approval of the manuscript. SWG, data collection, critical revision, and final approval of the manuscript. JXZ, data collection, manuscript writing, and final approval of manuscript. HJX, interpretation, critical revision, and final approval of the manuscript. ZGL, conception and design, data collection and analysis, financial support, manuscript writing, final approval, and responsibility for the manuscript. All authors read and approved the final manuscript.

## Supplementary Material

Additional file 1: Figure S1 DNA sequence analysis of the three different genotypes of the rs7044343 and rs10975514. (a) The TT genotype of rs7044343. (b) The CT genotype of rs7044343. (c) The CC genotype of rs7044343. (d) The GG genotype of rs10975514. (e) The AG genotype of rs10975514. (f) The AA genotype of rs10975514. Click here for file

Additional file 2: Table S1 Association analysis of rs10975514 with RA in the discovery population. **Table S2.** Association analysis of rs10975514 with RA in the validation population. **Table S3.** Association analysis of rs10975514 with RA in the replication population. Click here for file
