# Supplementary material for: Genetic variant in IL33 is associated with susceptibility to rheumatoid arthritis
Source: Arthritis Res Ther. 2014 Apr 29;16(2):R105. doi: 10.1186/ar4554 (PMC4075243; doi:10.1186/ar4554)
Supplement: Additional file 1: Figure S1 — DNA sequence analysis of the three different genotypes of the rs7044343 and rs10975514. (a) The TT genotype of rs7044343. (b) The CT genotype of rs7044343. (c) The CC genotype of rs7044343. (d) The GG genotype of rs10975514. (e) The AG genotype of rs10975514. (f) The AA genotype of rs10975514. [file ar4554-S1.pdf]

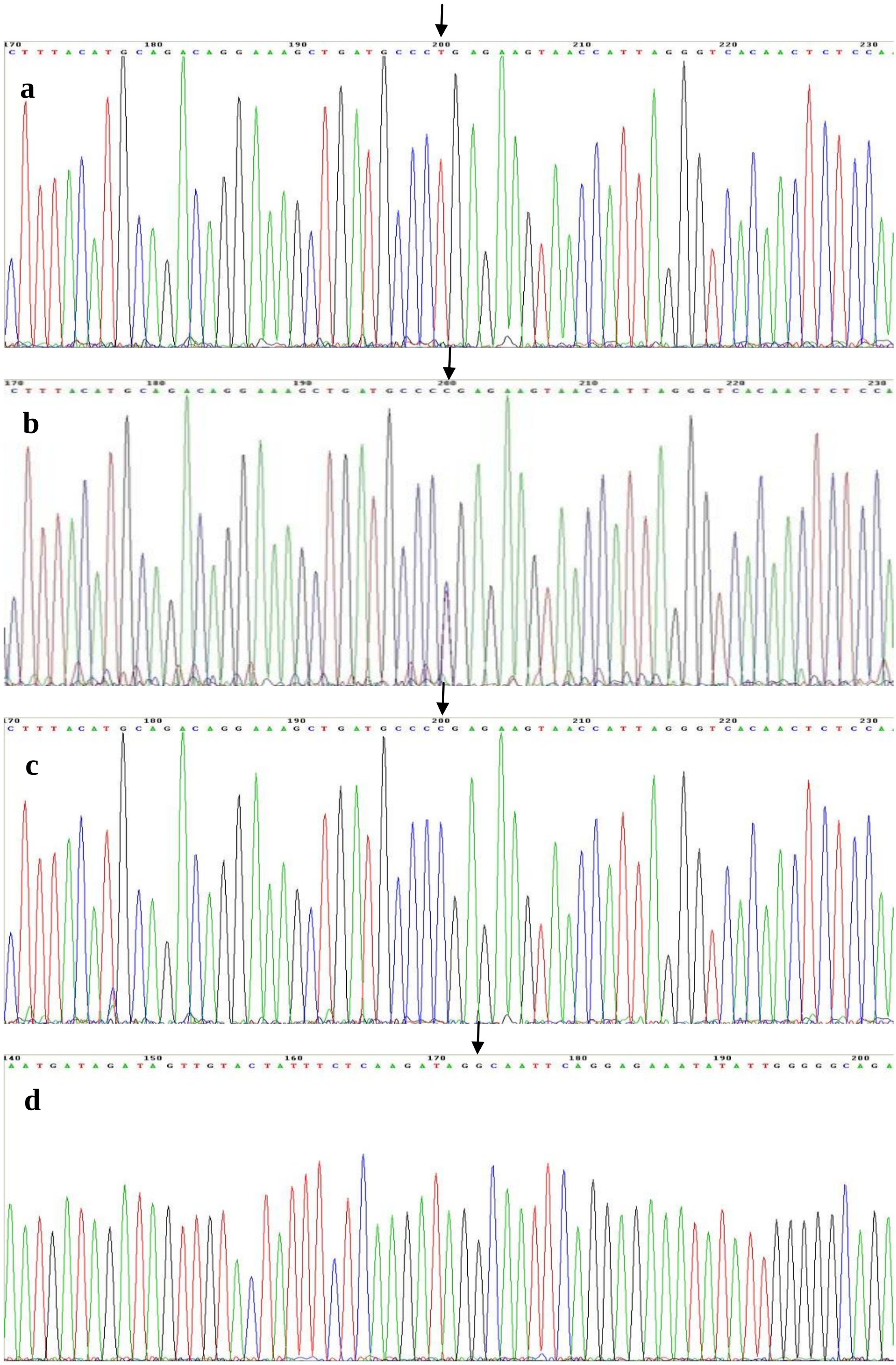
a
b
c
d

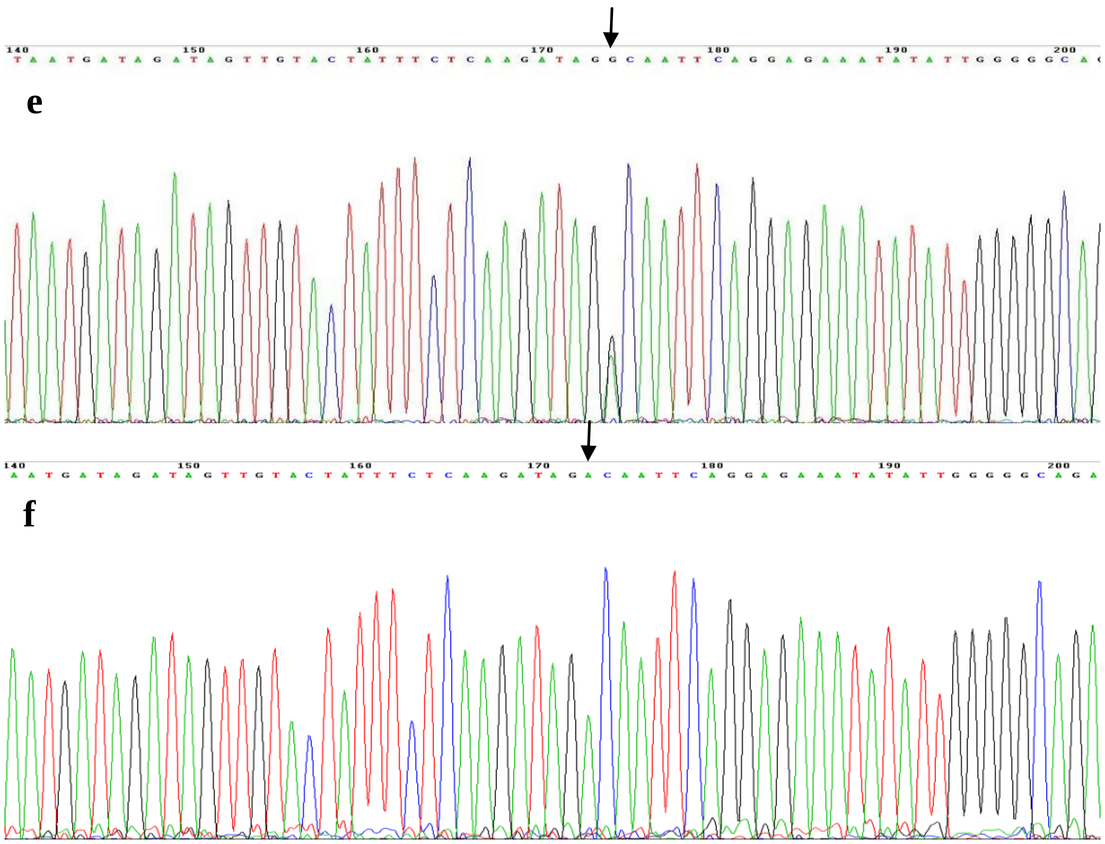


**Supplementary Figure 1. DNA sequence analysis of the three different genotypes of the rs7044343 and rs10975514.** a The TT genotype of rs7044343. b The CTgenotype of rs7044343. c The CC genotype of rs7044343. d The GG genotype of rs10975514. e The AG genotype of rs10975514. f The AA genotype of rs10975514.
