# Supplementary material for: Genetic variant in IL33 is associated with susceptibility to rheumatoid arthritis
Source: Arthritis Res Ther. 2014 Apr 29;16(2):R105. doi: 10.1186/ar4554 (PMC4075243; doi:10.1186/ar4554)
Supplement: Additional file 2: Table S1 — Association analysis of rs10975514 with RA in the discovery population. Table S2. Association analysis of rs10975514 with RA in the validation population. Table S3. Association analysis of rs10975514 with RA in the replication population. [file ar4554-S2.pdf]

Supplementary Table 1. Association analysis of rs10975514 with RA in the discovery population

| Characteristic (n) | Allele frequency(%) | | Genotype frequency(%) | | | A vs. G | AA vs. AG+GG | AA+AG vs. GG |
|---|---|---|---|---|---|---|---|---|
| | A | G | AA | AG | GG | OR(95%CI) P-value | OR(95%CI) P-value | OR(95%CI) P-value |
| CON (598) | 693 (57.9) | 503 (42.1) | 192(32.1) | 309 (51.7) | 97 (16.2) | | | |
| RA (700) | 818 (58.4) | 582 (41.6) | 235 (33.6) | 348 (49.7) | 117(16.7) | 1.020 (0.872-1.193) 0.811 | 1.069 (0.847-1.348) 0.576 | 0.965 (0.719-1.295) 0.811 |
| RF+ (418) | 490 (58.6) | 346 (41.4) | 143 (34.2) | 204 (48.8) | 71 (17.0) | 1. 028 (0.859-1.230) 0.763 | 1.100 (0.843-1.433) 0.483 | 0.946 (0.677-1. 323) 0.747 |
| RF– (84) | 95 (56.5) | 73 (43.5) | 23 (27.4) | 49 (58.3) | 12 (14.3) | 0.945 (0.682-1. 309) 0.732 | 0.797 (0.479-1.327) 0.383 | 1.162 (0.607-2.222) 0.651 |
| ACPA+ (246) | 282 (57.3) | 210 (42.7) | 75 (30.5) | 132 (53.7) | 39 (15.9) | 0.975 (0.788-1.205) 0.813 | 0.927 (0.673-1.279) 0.646 | 1.028 (0.685-1.541) 0.895 |
| ACPA– (84) | 101 (60.1) | 67 (39.9) | 29 (34.5) | 43 (51.2) | 12 (14.3) | 1.094 (0.787-1.521) 0.592 | 1.115 (0.689-1.804) 0.658 | 1.162 (0.607-2.222) 0.651 |
| Female CON (412) | 472 (57.3) | 352 (42.7) | 131 (31.8) | 210 (51.0) | 71(17.2) | | | |
| Female RA (511) | 596 (58.3) | 426 (41.7) | 169 (33.1) | 258 (50.5) | 84 (16.4) | 1.043 (0.866-1.256) 0.654 | 1.060 (0.803-1.399) 0.681 | 1.058 (0.748-1.497) 0.748 |
| Female RF+ (338) | 396 (58.6) | 280 (41.4) | 113 (33.4) | 170 (50.3) | 55 (16.3) | 1.055 (0.858-1.296) 0.612 | 1.077 (0.793-1.464) 0.634 | 1.071 (0.728-1.576) 0.726 |
| Female RF– (71) | 80 (56.3) | 62 (43.7) | 20 (28.2) | 40 (56.3) | 11 (15.5) | 0.962 (0.672-1.378) 0.834 | 0.841 (0.482-1.468) 0.543 | 1.136 (0.569-2.268) 0.718 |
| Female ACPA+ | 234(5 | 174 | 62 (30.4) | 110 (53.9) | 32 (15.7) | 1.003 | 0.937 | 1.119 |

| | | | | | | | | |
|---|---|---|---|---|---|---|---|---|
| (204) | 7.4) | (42.6) | | | | (0.789-1.275) 0.981 | (0.651-1.347) 0.724 | (0.709-1.765) 0.628 |
| Female ACPA– (67) | 82 (61.2) | 52 (38.8) | 24 (35.8) | 34 (50.7) | 9 (13.4) | 1.176 (0.809-1.709) 0.395 | 1.197 (0.697-2.056) 0.514 | 1.342 (0.636-2.833) 0.441 |
| Male CON (123) | 136 (55.3) | 110 (44.7) | 35 (28.5) | 66(53.7) | 22 (17.9) | | | |
| Male RA (115) | 130 (56.5) | 100 (43.5) | 37 (32.2) | 56 (48.7) | 22 (19.1) | 1.051 (0.732-1.510) 0.786 | 1.193 (0.686-2.075) 0.533 | 0.921 (0.478-1.772) 0.805 |
| Male RF+ (79) | 93 (58.9) | 65 (41.1) | 30 (38.0) | 33 (41.8) | 16 (20.3) | 1.157 (0.772-1.734) 0.479 | 1.539 (0.845-2.805) 0.159 | 0.858 (0.419-1.756) 0.675 |
| Male RF– (12) | 13 (54.2) | 11 (45.8) | 2 (16.7) | 9(75.0) | 1 (8.3) | 0.956 (0.412-2.217) 0.916 | 0.503 (0.105-2.412) 0.390 | 2.396 (0.294-19.535) 0.414 |
| Male ACPA+ (42) | 48 (57.1) | 36 (42.9) | 13(31.0) | 22(52.4) | 7 (16.7) | 1.078 (0.654-1.778) 0.767 | 1.127 (0.526-2.416) 0.758 | 1.089 (0.428-2.770) 0.858 |
| Male ACPA– (14) | 17 (60.7) | 11 (39.3) | 5 (35.7) | 7 (50.0) | 2 (14.3) | 1.250 (0.562-2.779) 0.584 | 1.397 (0.437-4.461) 0.573 | 1.307 (0.273-6.259) 0.738 |

*$P<0.05$ *vs* controls; RA, rheumatoid arthritis; CON, healthy controls; RF, rheumatoid factor; ACPA, anti-citrullinated proteins antibodies; OR, Odds ratio; CI, confidence interval.

Supplementary Table 2. Association analysis of rs10975514 with RA in the validation population

| Characteristic (n) | Allele frequency(%) | | Genotype frequency(%) | | | A vs. G | AA vs. AG+GG | AA+AG vs. GG |
|---|---|---|---|---|---|---|---|---|
| | A | G | AA | AG | GG | OR(95%CI) P-value | OR(95%CI) P-value | OR(95%CI) P-value |
| CON (456) | 524 (57.5) | 388 (42.5) | 149(32.7) | 226 (49.6) | 81 (17.8) | | | |
| RA (586) | 687 (58.6) | 485 (41.4) | 200 (34.1) | 287(49.0) | 99(16.9) | 1.049 (0.880-1.250) 0.594 | 1.068 (0.823-1.384) 0.622 | 1.063 (0.769-1.468) 0.713 |
| RF+ (357) | 426 (59.7) | 288 (40.3) | 125 (35.0) | 176 (49.3) | 56 (15.7) | 1. 095 (0.898-1.336) 0.370 | 1.110 (0.829-1.487) 0.484 | 1.161 (0.800-1. 686) 0.433 |
| RF– (104) | 118 (56.7) | 90 (43.3) | 35 (33.7) | 48 (46.2) | 21 (20.2) | 0.971 (0.716-1. 316) 0.849 | 1.045 (0.666-1.641) 0.848 | 0.854 (0.500-1.459) 0.563 |
| ACPA+ (312) | 379 (60.7) | 245 (39.3) | 113 (36.2) | 153 (49.0) | 46 (14.7) | 1.145 (0.931-1.410) 0.199 | 1.170 (0.864-1.583) 0.309 | 1.249 (0.842-1.853) 0.269 |
| ACPA– (60) | 63 (52.5) | 57 (47.5) | 17 (28.3) | 29 (48.3) | 14 (23.3) | 0.818 (0.559-1.199) 0.303 | 0.815 (0.449-1.476) 0.499 | 0.710 (0.372-1.352) 0.297 |
| Female CON (415) | 482 (58.1) | 348 (41.9) | 140 (33.7) | 202 (48.7) | 73(17.6) | | | |
| Female RA (411) | 477 (58.0) | 345 (42.0) | 136 (33.1) | 205(49.9) | 70 (17.0) | 0.998 (0.821-1.214) 0.986 | 0.971 (0.727-1.297) 0.844 | 1.040 (0.725-1.491) 0.832 |
| Female RF+ (286) | 340 (59.4) | 232 (40.6) | 98(34.3) | 144 (50.3) | 44 (15.4) | 1.058 (0.852-1.314) 0.609 | 1.024 (0.745-1.407) 0.884 | 1.174 (0.780-1.767) 0.442 |

| | | | | | | | | |
|---|---|---|---|---|---|---|---|---|
| Female RF–<br>(86) | 100<br>(58.1) | 72<br>(41.9) | 30 (34.9) | 40 (46.5) | 16 (18.6) | 1.003<br>(0.719-1.399)<br>0.987 | 1.052<br>(0.646-1.714)<br>0.838 | 0.934<br>(0.513-1.700)<br>0.823 |
| Female ACPA+<br>(252) | 302<br>(59.9) | 202<br>(40.1) | 86 (34.1) | 130 (51.6) | 36 (14.3) | 1.079<br>(0.862-1.352)<br>0.506 | 1.018<br>(0.731-1.416)<br>0.917 | 1.281<br>(0.830-1.977)<br>0.264 |
| Female ACPA–<br>(47) | 53<br>(56.4) | 34<br>(43.6) | 15 (31.9) | 23 (48.9) | 9(19.1) | 1.125<br>(0.716-1.769)<br>0.608 | 0.921<br>(0.483-1.757)<br>0.802 | 0.901<br>(0.418-1.945)<br>0.791 |
| Male CON<br>(41) | 42<br>(51.2) | 40<br>(48.8) | 9(22.0) | 24(58.5) | 8 (19.5) | | | |
| Male RA<br>(51) | 52<br>(51.0) | 50<br>(49.0) | 12 (23.5) | 28 (54.9) | 11 (21.6) | 0.990<br>(0.554-1.772)<br>0.974 | 1.094<br>(0.410-2.923)<br>0.858 | 0.882<br>(0.318-2.446)<br>0.809 |
| Male RF+<br>(36) | 39<br>(54.2) | 33<br>(45.8) | 9 (25.0) | 21 (58.3) | 6 (16.7) | 1.126<br>(0.597-2.123)<br>0.715 | 1.185<br>(0.412-3.409)<br>0.753 | 1.212<br>(0.377-3.899)<br>0.747 |
| Male RF–<br>(13) | 11<br>(42.3) | 15<br>(57.7) | 2 (15.4) | 7(53.8) | 4 (30.8) | 0.698<br>(0.287-1.701)<br>0.428 | 0.646<br>(0.121-3.463)<br>0.610 | 0.545<br>(0.133-2.231)<br>0.399 |
| Male ACPA+<br>(25) | 27<br>(54.0) | 23<br>(46.0) | 8(32.0) | 11(44.0) | 6 (24.0) | 1.118<br>(0.553-2.262)<br>0.756 | 1.673<br>(0.546-5.125)<br>0.367 | 0.768<br>(0.231-2.548)<br>0.666 |
| Male ACPA–<br>(9) | 6<br>(33.3) | 12<br>(66.7) | 0 (0) | 6 (66.7) | 3 (33.3) | 0.476<br>(0.163-1.390)<br>0.169 | 0.000<br>(0.000- )<br>0.999 | 0.485<br>(0.099-2.370)<br>0.371 |

*$P$<0.05 *vs* controls; RA, rheumatoid arthritis; CON, healthy controls; RF, rheumatoid factor; ACPA, anti-citrullinated proteins antibodies; OR, Odds ratio; CI, confidence interval.

Supplementary Table 3. Association analysis of rs10975514 with RA in the replication population

| Characteristic (n) | Allele frequency(%) | | Genotype frequency(%) | | | A vs. G | AA vs. AG+GG | AA+AG vs. GG |
|---|---|---|---|---|---|---|---|---|
| | A | G | AA | AG | GG | OR(95%CI) P-value | OR(95%CI) P-value | OR(95%CI) P-value |
| CON (701) | 830 (59.2) | 572 (40.8) | 266(37.9) | 298(42.5) | 137 (19.5) | | | |
| RA (666) | 804 (60.4) | 528 (39.6) | 248(37.2) | 308(46.2) | 110(16.5) | 1.049 (0.901-1.223) 0.537 | 0.970 (0.779-1.208) 0.787 | 1.228 (0.931-1.620) 0.146 |
| RF+ (327) | 393 (60.1) | 261 (39.9) | 123 (37.6) | 147 (45.0) | 57 (17.4) | 1.038 (0.859-1.254) 0.702 | 0.986 (0.752-1.293) 0.919 | 1.151 (0.818-1. 619) 0.420 |
| RF– (61) | 72 (59.0) | 50 (41.0) | 20 (32.8) | 32 (52.5) | 9 (14.8) | 0.992 (0.681-1. 446) 0.968 | 0.798 (0.458-1.391) 0.426 | 1.403 (0.675-2.918) 0.364 |
| ACPA+ (221) | 269 (60.9) | 173 (39.1) | 82 (37.1) | 105 (47.5) | 34 (15.4) | 1.072 (0.861-1.334) 0.536 | 0.965 (0.706-1.319) 0.822 | 1.336 (0.886-2.014) 0.166 |
| ACPA– (41) | 48 (58.5) | 34 (41.5) | 15 (36.6) | 18 (43.9) | 8 (19.5) | 0.973 (0.619-1.529) 0.905 | 0.943 (0.491-1.814) 0.861 | 1.002 (0.453-2.218) 0.996 |
| Female CON (507) | 614 (60.6) | 400 (39.4) | 203 (40.0) | 208 (41.0) | 96(18.9) | | | |
| Female RA (461) | 541 (58.7) | 381 (41.3) | 162 (35.1) | 217(47.1) | 82 (17.8) | 0.925 (0.771-1.109) 0.401 | 0.811 (0.625-1.053) 0.116 | 1.080 (0.779-1.496) 0.645 |
| Female RF+ (263) | 311 (59.1) | 215 (40.9) | 95(36.1) | 121(46.0) | 47 (17.9) | 0.942 (0.760-1.168) 0.588 | 0.847 (0.622-1.152) 0.290 | 1.073 (0.730-1.579) 0.719 |
| Female RF– (52) | 61 (58.7) | 43 (41.3) | 17 (32.7) | 27 (51.9) | 8 (15.4) | 0.924 (0.613-1.393) 0.706 | 0.727 (0.397-1.333) 0.303 | 1.285 (0.586-2.818) 0.532 |
| Female ACPA+ | 205 | 147 | 58 (33.0) | 89 (50.6) | 29 (16.5) | 0.909 | 0.736 | 1.184 |

| | | | | | | | | |
|---|---|---|---|---|---|---|---|---|
| (176) | (58.2) | (41.8) | | | | (0.710-1.162)<br>0.445 | (0.513-1.056)<br>0.096 | (0.750-1.868)<br>0.468 |
| Female ACPA–<br>(35) | 42<br>(60.0) | 28<br>(40.0) | 13 (37.1) | 16 (45.7) | 6(17.1) | 0.977<br>(0.596-1.602)<br>0.927 | 0.885<br>(0.436-1.797)<br>0.735 | 1.129<br>(0.456-2.795)<br>0.793 |
| Male CON<br>(120) | 136<br>(56.7) | 104<br>(43.3) | 42(35.0) | 52(43.3) | 26 (21.7) | | | |
| Male RA<br>(96) | 127<br>(66.1) | 65<br>(33.9) | 45 (46.9) | 37 (38.5) | 14 (14.6) | 1.494<br>(1.008-2.214)<br>0.045 | 1.639<br>(0.946-2.837)<br>0.078 | 1.620<br>(0.793-3.309)<br>0.185 |
| Male RF+<br>(54) | 73<br>(67.6) | 35<br>(32.4) | 27 (50.0) | 19 (35.2) | 8 (14.8) | 1.595<br>(0.990-2.570)<br>0.054 | 1.857<br>(0.967-3.565)<br>0.063 | 1.590<br>(0.668-3.786)<br>0.294 |
| Male RF–<br>(9) | 11<br>(61.1) | 7<br>(38.9) | 3 (33.3) | 5(55.6) | 1 (11.1) | 1.202<br>(0.450-3.206)<br>0.713 | 0.929<br>(0.221-3.903)<br>0.919 | 2.213<br>(0.265-18.504)<br>0.464 |
| Male ACPA+<br>(44) | 64<br>(72.7) | 24<br>(27.3) | 24(54.5) | 16(36.4) | 4 (9.1) | 2.039<br>(1.195-3.479)<br>0.008 | 2.229<br>(1.104-4.497)<br>0.025 | 2.766<br>(0.906-8.442)<br>0.074 |
| Male ACPA–<br>(6) | 6<br>(50.0) | 6<br>(50.0) | 2 (33.3) | 2 (33.3) | 2 (33.3) | 0.765<br>(0.240-2.439)<br>0.650 | 0.929<br>(0.163-5.282)<br>0.933 | 0.553<br>(0.096-3.190)<br>0.508 |

*$P$<0.05 *vs* controls; RA, rheumatoid arthritis; CON, healthy controls; RF, rheumatoid factor; ACPA, anti-citrullinated proteins antibodies; OR, Odds ratio; CI, confidence interval.
